# The biology of *Marchantia polymorpha* subsp*. ruderalis* Bischl. & Boissel. Dub in nature

**DOI:** 10.3389/fpls.2024.1339832

**Published:** 2024-05-30

**Authors:** Jeffrey G. Duckett, Silvia Pressel, Jill Kowal

**Affiliations:** ^1^ Research, The Natural History Museum, London, United Kingdom; ^2^ Department of Ecosystem Stewardship, Royal Botanic Gardens Kew, London, United Kingdom

**Keywords:** carpocephala, female bias, fertilization distances, post-fire establishment, sex ratio, spore viability

## Abstract

**Introduction:**

Though used as the model liverwort in culture for several decades, the biology of Marchantia polymorpha subsp. ruderalis in nature has never been documented in detail in a single account.

**Methods:**

Here we synthesize routine field observations documented with hundreds of images of M. ruderalis colonies (or groups) showing sex differentiation over 3 years on two populations of M. ruderalis after major heathland fires in 2020.

**Results:**

Initial post-fire establishment is from airborne spores rather than a spore bank but thereafter spread is via gemmae which have less exacting germination requirements. Young sporelings are highly gemmiferous but gemmae production becomes less frequent after sex organ formation. Over the course of a year there are up to three waves of carpocephalum production with the overwhelming majority of antheridiophores appearing 2-3 months ahead of the archegoniophores though no differences in growth rates were apparent between male and female thalli. Spermatozoids are produced almost continuously throughout the year, whilst sporophyte maturation is restricted to the summer months.

**Discussion:**

Because of the asynchrony between antheridiophore and archegoniophore production a 1:1 sex ratio is only apparent over this period. The spring months see an excess of males with more females in the summer. An almost 100% fertilization rate, with fertilization distances of up to 19 m far exceeding those in all other bryophytes, is attributed to vast spermatozoid production for most of the year, dispersal on surface oil films between thalli and highly effective intra-thallus spermatozoid transport via the pegged-rhizoid water-conducting system. Archegoniophores do develop on female-only populations but have shorter stalks than those where fertilization has occurred. Eventual disappearance post fires is attributed to a fall in topsoil nutrient levels preventing new sporeling establishment and competition from Ceratodon purpureus and Polytrichum spp. A major drought in the summer of 2022 almost wiped out the heathland Marchantia populations but all the other bryophytes survived.

## Introduction

*Marchantia polymorpha* subsp*. ruderalis* Bischl. & Boissel. Dub. is a Circumboreal-temperate cosmopolitan liverwort found throughout Europe and in all continents except Antarctica (Blockeel et al., 2014). Other than describing it as a species of disturbed habitats, particularly after fires and man-made, where it can become invasive e.g., between paving stones in urban streets and in greenhouses, floras and field guides provide almost no information on its actual biology and longevity. Paton (1999) reports that sporophytes are uncommon but makes no mention of the time of year. Quoting from Duckett and Pressel (2009); Blockeel et al. (2014) state that carpocephala (gametangiophores) may be stimulated by long days and fairly high temperatures and that gemmae are always present. *M. polymorpha* subsp. *polymorpha* L. and subsp. *montevagans* Bischl. & Boissel. Dub. occur in more permanent habitats and appear to be less fertile than subsp. *ruderalis* (Paton, 1999).

**Figure 1:**
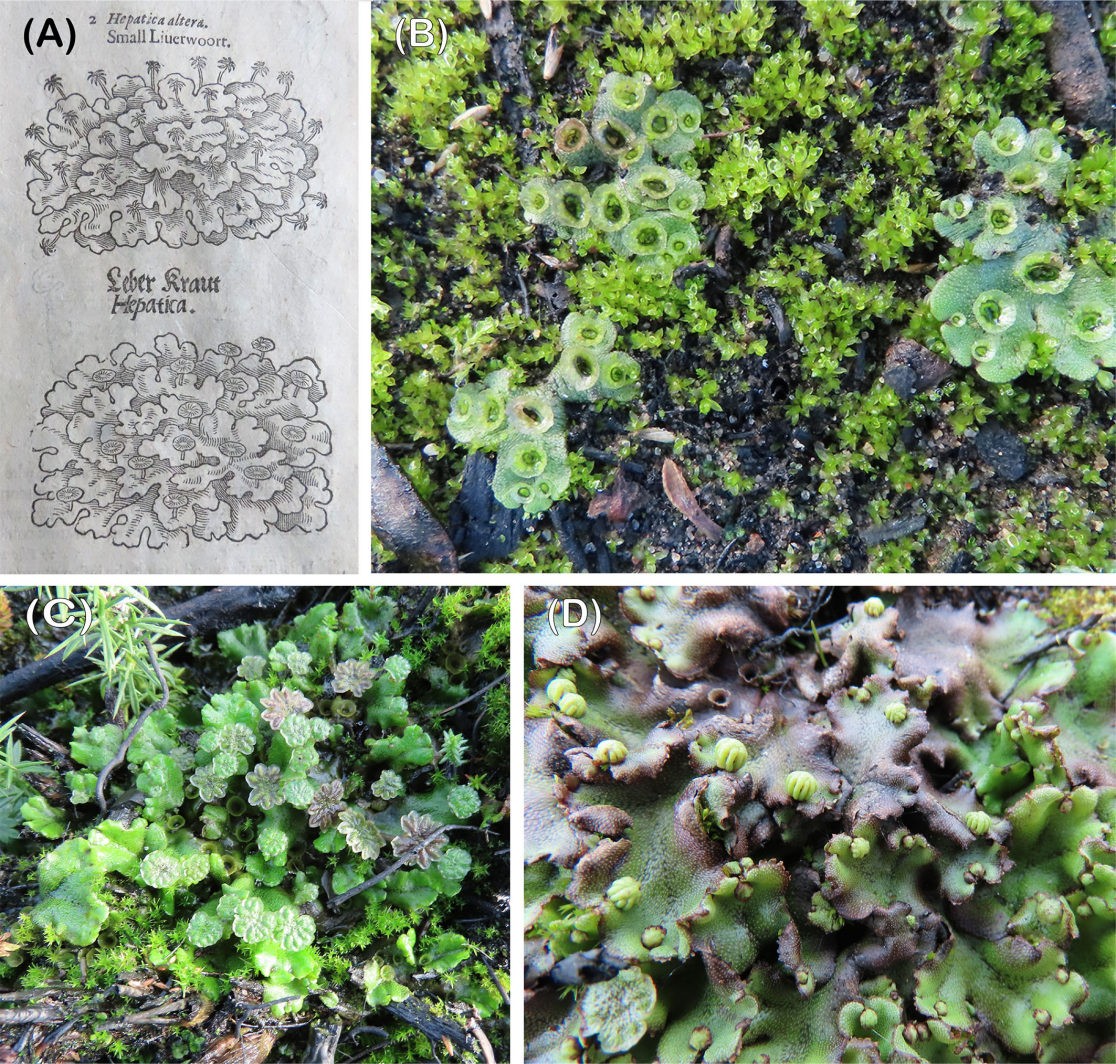
**(A)** One of the earliest illustrations of male and female *Marchantia*: Gerarde’s Herbal, 1597, **(B)** highly gemmiferous sporelings growing with young *Funaria*. Thursley, October 2020, **(C)** male colony with three generations of antheridiophores; old, mature and young. Thursley, December 2020, **(D)** young archegoniophores. Thursley, April 2021.

For more than four centuries *Marchantia polymorpha* has figured as the archetypal liverwort in textbook descriptions (**Figure 1A**) (Gerarde, 1597). With wild material readily available, ease of cultivation and amenability to biochemical and molecular analyses it is unsurprising therefore that today *Marchantia* is the model liverwort (Shimamura, 2016; Bowman et al., 2022), alongside the hornwort *Anthoceros agrestis* (Szövényi et al., 2015; Frangedakis et al., 2021) and the mosses *Ceratodon purpureus* (Shaw and Gaughan, 1993; Kollar et al., 2021), *Funaria hygrometrica* and *Physcomitrium paten*s (Wood et al., 2000). Wild collections are the usual starting point for advanced research programmes on these model species except for *Physcomitrim patens* where the Gransden strain, isolated from a single spore in 1959, continues to be widely used despite its reduced fertility (Rensing et al., 2020).

In striking contrast to the vast body of research on cultured plants, the behaviour of these bryophytes in nature remains very poorly documented except perhaps for the reproductive cycles of *Funaria* (Duckett and Pressel, 2022) and to a lesser extent of *Ceratodon* (Kollar et al., 2021; Duckett et al., 2023). The wild reproductive biology of *Marchantia* from initial establishment to its disappearance has never been set out systematically in a single account though snapshots of particular phases have been reported in popular articles e.g., remarkable fertilization distances (Pressel and Duckett, 2019), mammoth sporophyte production (Duckett and Pressel, 2009), asynchrony between antheridiophore and archegoniophore formation (Pressel et al., 2021; Duckett et al., 2023), a dramatic decline in gemmae production with thallus age (Duckett et al., 2023) and extinction following drought (Duckett et al., 2023). These reveal that the biology of *Marchantia* in nature is far more complicated than might appear at first sight.

Reported here is a synthesis of over three years of field data collected at approximately monthly intervals on *Marchantia ruderalis* following fires in the summer of 2020 at two lowland heaths in Southern England plus other incidental information on other populations across southern England. We hope that this will serve as a foundation reference work for future molecular studies.

## Sites and methods

**Figure 2:**
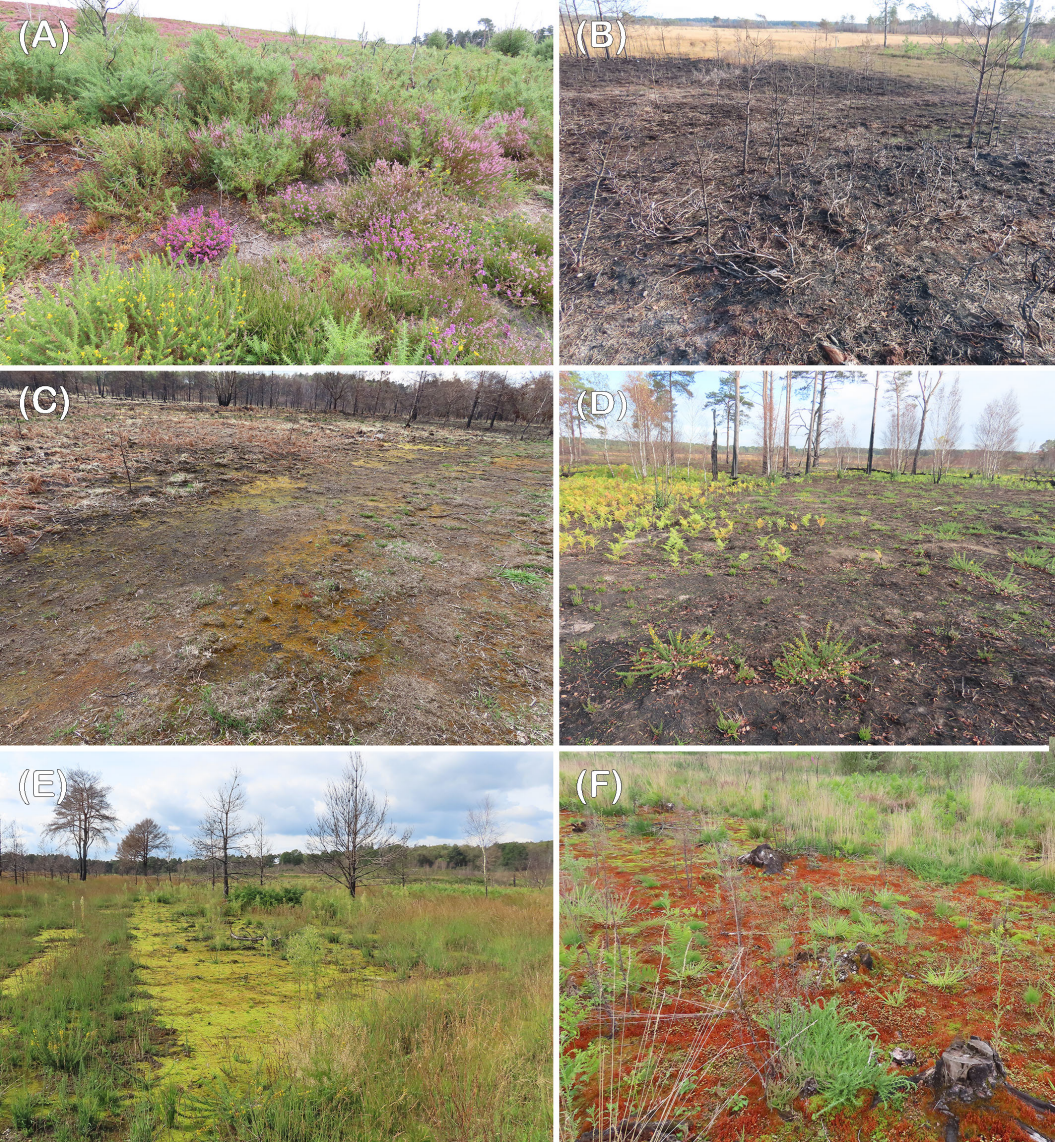
**(A, B, D)** Thursley, **(C, E, F)** Chobham. **(A)** Unburnt area with *Erica*, *Calluna* and *Ulex* sp., **(B)** Burnt area prior to recolonisation, December 2020, **(C, D)** later recolonisation stages showing different degrees of bryophyte coverage, late spring to autumn 2021, **(E, F)** almost continuous carpets of *Funaria* and *Ceratodon*, 2022.

Thursley Common, National Nature Reserve and Chobham Common (both Sites of Special Scientific Interest) are major lowland heath nature reserves in southern England with extensive areas of open dry and wet heathland, peat bogs, pine and deciduous woodlands. They are renowned for dragonfly and bird diversity. Periodically they are subjected to major fires, the last prior those in 2020 being at Thursley in 2006 (Duckett et al., 2008). Post the fires the areas recolonized by *Marchantia* were previously dominated by Ericaceae; *Calluna vulgaris* and *Erica cinerea* on dry heath, *Erica tetralix* in wetter areas. Full species lists and details of post fire recolonization are given in Pressel et al. (2021) and Duckett et al. (2023), see also **Figure 2**.

Both sites have the same mild Atlantic climate. Monthly mean temperatures range from 18 degrees in the summer months down to 7 degrees in the winter. Monthly rainfall is between 65 and 85 mm throughout the year. There are light frosts (-1 to -5 degrees) between 30 and 45 days but almost no days when temperatures persist below zero for 24h. Apart from the short frosts and periods of up to 4 weeks without rain, in spring and summer, *Marchantia* grew continuously throughout the year at both study sites.

The sites were visited at approximately monthly intervals since the fires. On each visit over 100 images were taken of groups of *Marchantia* colonies. These were subsequently analysed for the numbers of male, females, and vegetative individuals. The presence of sporophytes on mature archegoniophores on at least 20 individuals was noted in the field.

More incidental observations were made on ten other *Marchantia* populations across southern England.

## Observations and Discussion

### Initial colonization

*Marchantia* first appeared at Thursley some 100 days after the 2020 fire as very numerous small thalli scattered over an area of several hectares. Only a small fraction of seemingly suitable burnt areas were colonized. It was very frequent in damper areas previously containing *Erica tetralix* and *Molinia caerulea* with an understory of *Polytrichum* species, *Sphagnum compactum, Campylopus paradoxus* and *C. brevipilus* but was absent in drier areas normally dominated by *Calluna vulgaris* and with *Campyoplus introflexus* and *Cephaloziella divaricata*.

Some of the 2020 burnt areas had also been affected by the earlier fire of 2006 (Duckett et al., 2008). However, after the 2006 fire *Marchantia* had a much wider and more even distribution than in 2020, enabling the measurement of fertilization distances (Pressel and Duckett, 2019). The most likely explanation for the more restricted distribution in 2020 was a dry period from April to June.

Long term spore survival in bryophytes is best documented in *Sphagnum* where viable spores can persist *in situ* for several decades (Clymo and Duckett, 1986; Sundberg and Rydin, 2000). There are numerous accounts of viability being retained for many years in herbarium specimens and the long term survival of moss spores, particularly of ephemeral and annual taxa (Furness and Hall, 1981; Jonsson, 1993). On the basis of extensive experimental data, During (1997) considers bryophyte spore banks to closely parallel those in seeds. We are not however aware of any studies specific to *Marchantia* and very few authors even mention liverworts. Van Zanten and Gradstein (1988), in their wide ranging appraisal of dispersal geography, follow Inoue (1961) by stating that *Marchantia* spores are likely to remain viable *in situ* for about a year or more whereas taxa that experience seasonal droughts (e.g., *Fossombronia, Riccia*) are likely to be much longer lived. Thus, we conclude that the post 2020 fire *Marchantia* at Thursley almost certainly came from airborne spores rather than those lying dormant in the soil since the previous fire in 2006. We now require measurements of the bryophyte spore rain at our two sites like those in Pettersson (1940).

Subsequent to the initial establishment event, new thalli grew clustered around existing ones with numbers declining radially with distance, in line with recorded distances of gemmae splash dispersal not exceeding 1.2 m (Stieha et al., 2014). This strongly suggests that secondary colonization, following initial establishment, occurred from gemmae rather than spores. A spore rain, either blown in or locally produced, should be excluded, as it would have produced a more uniform distribution. Establishment from spores appears to require much more exacting nutrient requirements e.g., high nitrogen as available immediately after fire, than those suitable for gemmae and is thus the most critical phase in the ecology of *Marchantia.* Needed now is precise information, particularly on soil surface N, P and K, post fires and comparisons with nutrient levels in culture media.

All the thalli on the two heaths were initially highly gemmiferous and vegetative (**Figure 1B**). Once the first gametangiophores began to appear 186 days from the fire, the thalli had become progressively less gemmiferous (Pressel et al., 2021). Our observations that urban pavement populations are invariably highly gemmiferous, point to similar proliferation via gemmae with sporeling establishment a much rarer event. In addition, the facts that these populations are almost always vegetative, or have one sex only, are further hallmarks of initial establishment from a single spore. On the other hand, single sex clusters of between 20 to over 200 individual plants after the 2006 (Pressel et al., 2021) and 2020 fires point to secondary origins from gemmae.

### Sexual cycles

**Figure 3:**
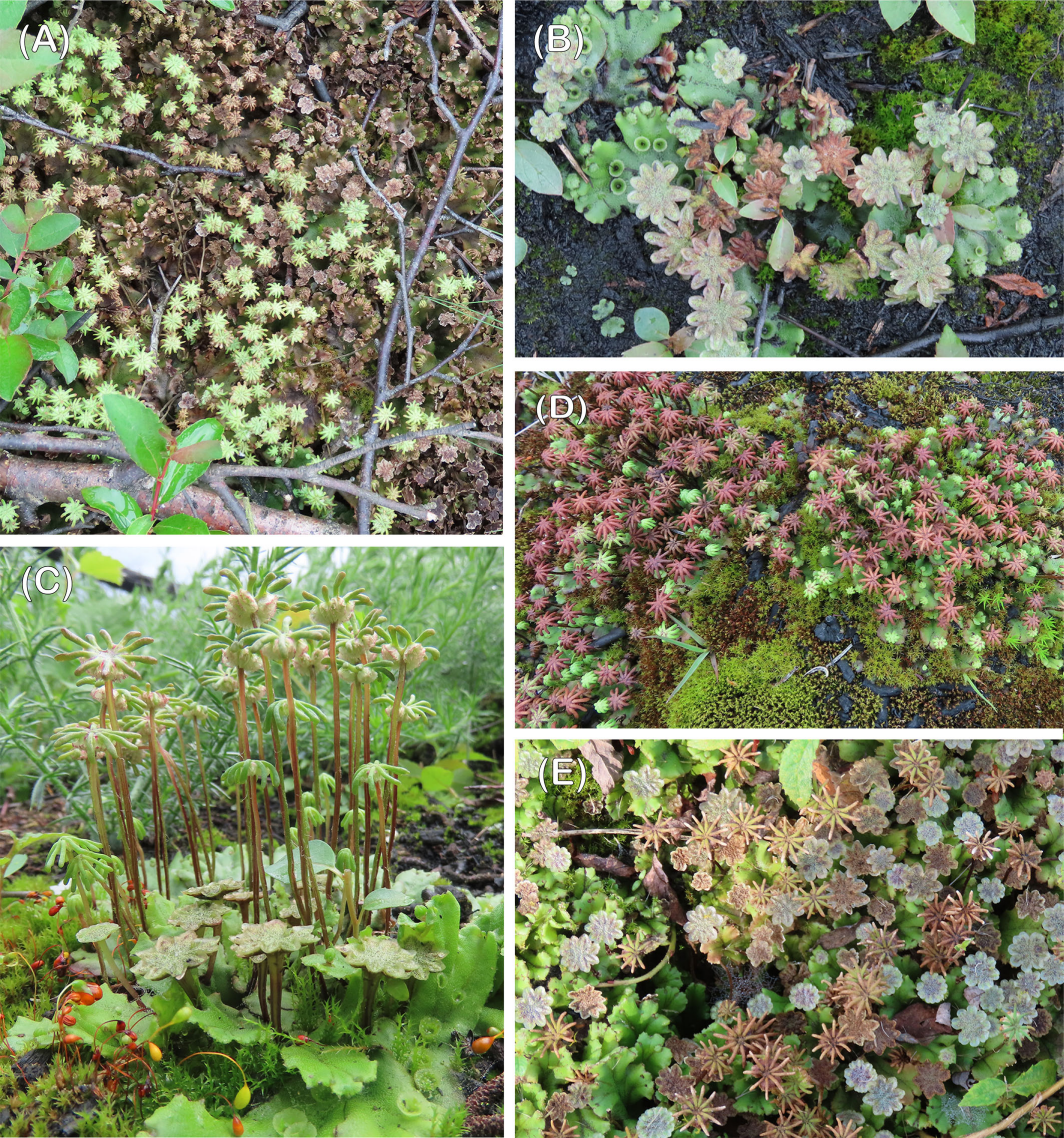
**(A)** Mature archegoniophores and old antheridiophores. Chobham, June 2021, **(B)** male colony with three generations of antheridiophores; old, mature and young. Chobham, June 2020, **(C)** mature male and female alongside *Funaria* with dehisced capsules. Chobham, July 2021, **(D)** female colonies with old and young archegoniophores. Thursley, August 2021, **(E)** old females and young males. Chobham, September 2021.

**Figure 4:**
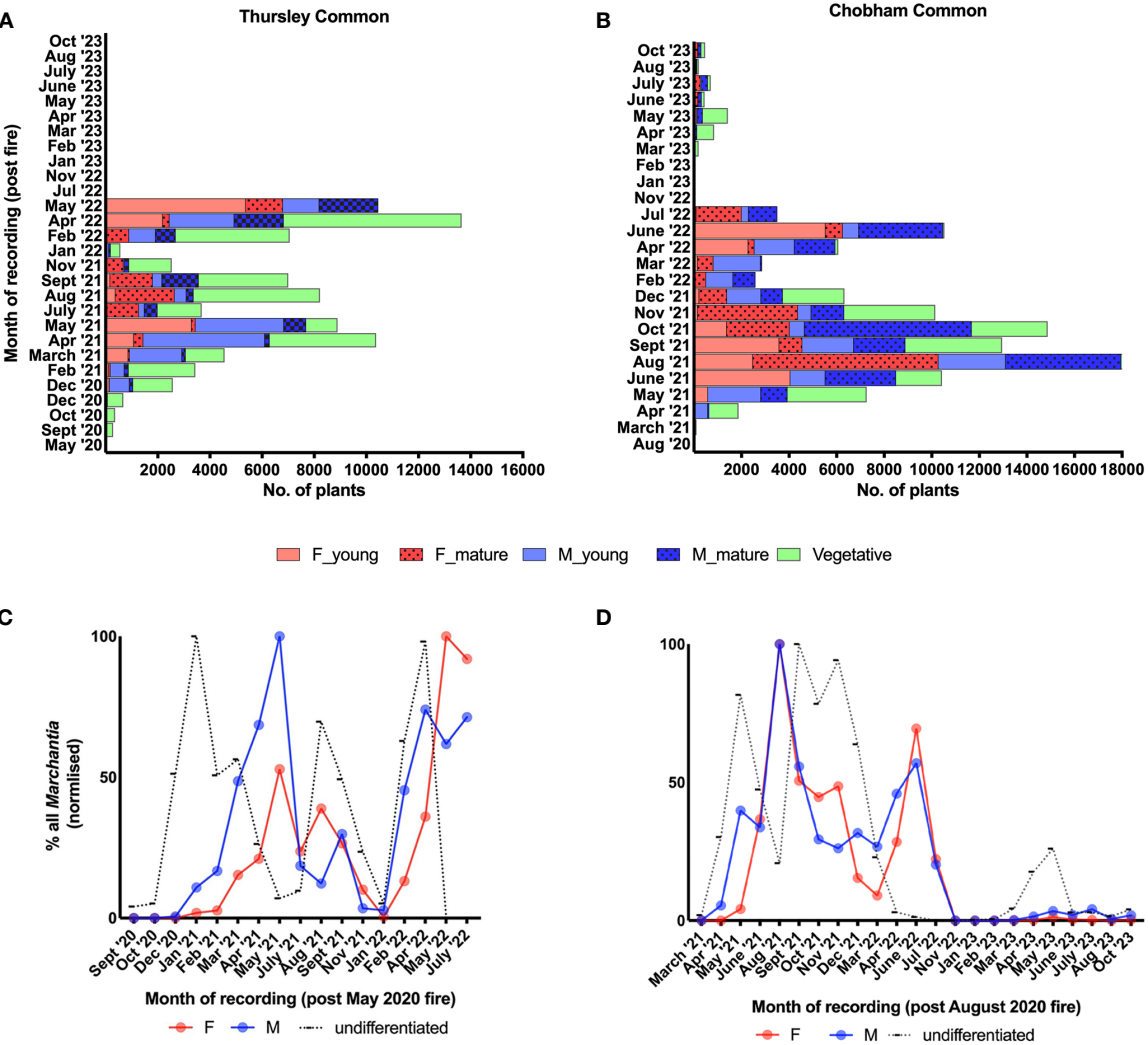
Seasonal changes in reproduction **(A, B)** and sex ratios - male (M), female (F) and undifferentiated **(C, D)**, in *Marchantia polymorpha* subsp*. ruderalis* populations after wildfires (in May and August 2020) at two different heathlands in Southern England, Thursley National Nature Reserve and Chobham Common, respectively. Note these follow similar patterns at both sites (Thursley – **A, C**; Chobham – **B, D**) and across 2 1/2 years until the complete disappearance at Thursley and scarce re-emergence at Chobham associated with drought in July 2022.

The first gametangiophores to develop in the winter months of 2020/21 were exclusively male (**Figure 1C**). Archegoniophores appeared nearly 2-3 months later (**Figure 1D**) with a further 2-3 months until spore dispersal in the summer (**Figures 3A, C**). 2021 saw two further cycles of sex organ production (**Figures 3B, D**) and this pattern was repeated in subsequent years at both sites. At all times except in midsummer (**Figure 3C**) antheridiophore and archegoniophore production are always out of step (**Figures 3A, E**). The summer cycles from archegoniophore initiation until spore dispersal were as low as 60 days to over double this time during the other seasons. These seasonal changes are summarised in **Figures 4A, B**.

Sperm discharge occurs continuously throughout the year except over late summer and early winter (August to December), whereas mature archegonia are absent from late autumn until spring (September until March). We have noted spore discharge from late May until October but peaking in July and August. There is never a time of year when maturing archegonia lack a supply of spermatozoids. Although midsummer counting of male and female thalli produces, at first sight, a roughly 1:1 sex ratio, because of the temporal differences between male and female maturation times set out above, this is a gross oversimplification (**Figures 4C, D**). After initial establishment most of the vegetative thalli are those where gametangiophores have decayed and disappeared.

Given the multiple cycles of gametangiophore production per annum (**Figures 1C**, **3B, D**), prevailing temperature conditions, rather than day length, are the most likely trigger, assuming sufficient rain to keep the thalli hydrated. We found no evidence for any significant dormant period, cf. *Lunularia cruciata* (Duckett and Pressel, 2023) just faster and slower periods of thallus growth. *Funaria* also has several life cycles per year in striking contrast to the production of spores once a year in *Campylopus introflexus* and *Ceratodon* (Duckett et al., 2023) or 16 months in *Polytrichum* (Duckett and Pressel, 2023).

**Figure 5:**
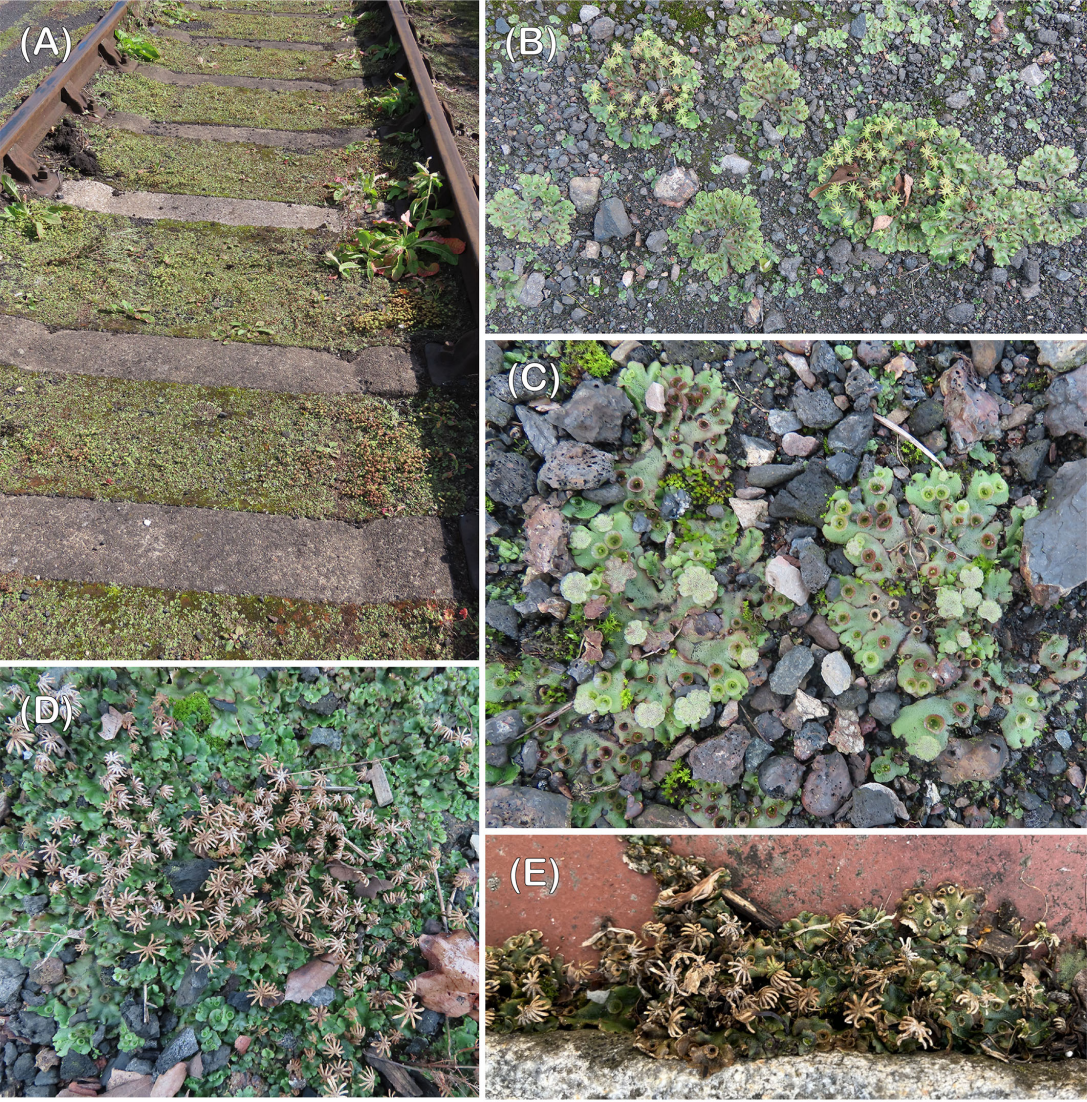
**(A)** Huge population of *Marchantia polymorpha* subsp*. ruderalis* on clinker along an old railway line. Didcot Oxfordshire, England, August 2023, **(B)** railway clinker colonies August 2023; sporophyte production by all the females indicates that the vegetative colonies are males that have lost their antheridiophores, **(C, D)** the railway clinker colonies, October 2023 – young males **(C)** and females with dead archegoniophores **(D)**, **(E)** female only pavement population; note the shorter archegoniophore stalks compared with those that have produced sporophytes (**Figure 1C**).

The constancy of this reproductive cycle over three years and at two different sites indicates this to be typical of post fire *Marchantia ruderalis* populations. Other wild populations in other habitats behave similarly with only antheridiophores present in the winter months. The most extreme example of temporal disparity between the sexes we have so far encountered is a huge population of tens of thousands of thalli on clinker between old railway lines at Didcot Railway Centre, southern England (**Figure 5A**). In August 2023 all the hundreds of sexual thalli we observed were female and we did not see a single antheridiophore, young, mature or moribund. However, the fact that all the females were also producing numerous sporophytes like those at Thursley and Chobham indicates that many of the vegetative thalli also present were males that had since lost their antheridiophores (**Figure 5B**). As expected, by October 2023, males with young antheridiophores (**Figure 5C**) were growing alongside females with long dead archegoniophores (**Figure 5D**). The only archegoniophores lacking sporophytes that we have seen have been on small single sex urban street populations (**Figure 5E**). The only difference between these and fertilised ones is shorter carpocephala stalks. Thus, archegoniophore maturation is not dependent on fertilization (Bisher, 1998) as considered by some earlier authors e.g., Goebel (1905).

Analysis of 100’s of digital field images revealed no differences between the diameters of male and female colonies, but female thalli tended to be more branched while males produced fewer gemmae and fewer antheridiophores. Ten cm diameter male colonies typically bore around 10 antheridiophores compared with 15-20 archegoniophores produced by same size female colonies. A parallel study of *Marchantia inflexa* populations in the Southern USA and the Caribbean (Stieha et al., 2014) also details earlier and more profuse gemmae production by males.

Following spermatozoid discharge, old antheridiophores can persist for 2-3 months while, after spore dispersal, old dead archegoniophores can still be found more than 6 months later. Vegetative thalli with few gemmae are almost certainly ones where gametangiophores had decayed previously rather than juvenile individuals.

Our documentation of sexual dynamics in *Marchantia* has major implications for working out sex ratios in bryophytes generally. These are typically female biased with explanations ranging from environmental factors to sex-specific traits (Longton and Schuster, 1983; McLetchie, 1992; Shaw and Gaughan, 1993; Bowker et al., 2000; McLetchie and Puterbaugh, 2000; Crowley et al., 2005; Bisang et al., 2006; Cronberg et al., 2006, 2015; Rydgren et al., 2006; Hedenäs et al., 2010; Horsley et al., 2011, 2010; Stark et al., 2010; Baughman et al., 2017; Bisang and Glime, 2017). In *Marchantia* this female bias has been reported in diverse locations, from open habitats in the Falkland Islands (*M. berteroana*, Engels, 1980; Duckett et al., 2012) to tropical forests in Puerto Rico (*M. chenopoda*, Moyá, 1992) and Trinidad (*M. inflexa*). In *M. inflexa* this is attributed to a faster growth rate and lower gemma production by females (Stieha et al., 2014). This bias was not apparent at Thursley nor at Chobham, where female favouritism is highly restricted to a few months in the summer. The female bias in the Falklands may simply be due to the fact that all the collections were in the summer months. In a study of sex ratios in Costa Rican *Marchantia* sp. populations, Garrison (2007) recorded an increasingly strong female metapopulation bias during the rainy season and suggested this might be due to higher male nutrient requirements.

Manipulating day length in green house grown plants Wann (1925) found that long days favour gametangiophore production in *Marchantia.* His discovery that antheridiophores appear after 4-6 weeks and archegoniophores only after 6-8 weeks fits closely with our field observations. Voth and Hamner (1940) also reported a greater number of gametangiophores under a long photoperiod than comparable plants on short photoperiod but did not record a temporal disparity between antheridiophore and archegoniophore formation.

Pointing out a general ignorance of the role of a circadian clock in daylength measurement in basal land plants Kanesaka et al. (2023) found a wildtype accession of *Marchantia polymorpha* (Takaragaike-1) to be an obligate long-day plant with a critical daylength of about 10 hours and requiring multiple long days for gametangiophore production. They then explored the timing of gametangiophore formation under various non-24-h light/dark cycles to examine the effect of phase alteration in circadian rhythms. They found that daylength measurement in *Marchantia* is based on the relative amounts of light and darkness rather than the intrinsic rhythms generated by a circadian clock. Thus, *Marchantia* may have a daylength measurement system different from that of angiosperms that centres on a circadian clock function.

Unfortunately, Kanesaka et al. (2023) only used male plants. Our field data strongly point to a long day requirement for archegoniophore formation from spring until early autumn but that this is highly unlikely for the antheridiophores as these are produced throughout the winter months with daylength down to 7h50min at the solstice. Kanesaka et al.’s work clearly requires repeating with females. It should be highlighted however, that the earlier production of antheridiophores ensures an abundant supply of spermatozoids to the archegoniophores and explains the abundant production of sporophytes in *Marchantia* (Duckett and Pressel, 2009). Whether this is the rule across dioicous bryophytes invites investigation. Antheridium production certainly precedes the formation of female shoots in monoicous *Funaria hygrometrica* (Duckett et al., 2023).

Needed now are *in vitro* studies comparing male and female growth rates and an analysis of the energetic costs of producing antheridiophores versus archegoniophores. This is of particular interest as stalked antheridiophores are restricted to just *Marchantia* and *Neohodgsonia* (Bischler-Causse et al., 1995). Sex-specific PCR assays (Iwasaki et al., 2021) are also needed to provide definitive proof of our assumption of a one:one sex ratio in vegetative thalli of *Marchantia ruderalis.*

### Remarkable fertilization distances and prolific sporophyte production

Our recording of fertilization distances of up to 19 m at Thursley in 2007 far exceeds those for all other bryophytes (see Pressel and Duckett, 2019 for full listings). Moreover, whatever the distance to the nearest male, every perianth on every archegoniophore produced a sporophyte which then went on to produce spores. This extremely high fertilization rate we attribute to: 1. The continuous production of huge numbers of spermatozoids. Each antheridium of *Marchantia* contains over 250,000 spermatozoids, far more than in any other land plant (Pressel and Duckett, 2019). 2. Long distance sperm dispersal between thalli on surface water films via oil droplets liberated from the antheridia (Muggoch and Walton, 1942). 3. Highly effective intra-thallus sperm transport both from the antheridiophores and into the archegoniophores via the peg rhizoid system (Duckett et al., 2013). 4. Resistance of the thalli to dehydration. Whereas on at least half our field visits in the summer months the mosses on the heaths were clearly dried up and desiccated, we only saw dehydrated *Marchantia* thalli following an extreme drought in the summer of 2022, although these, differently from the mosses, never recovered. The sight of fully hydrated *Marchantia* gametangiophores alongside dehydrated mosses, including *Polytrichum* spp. is most striking (see Duckett et al., 2013 for images).

### Post fire disappearance

**Figure 6:**
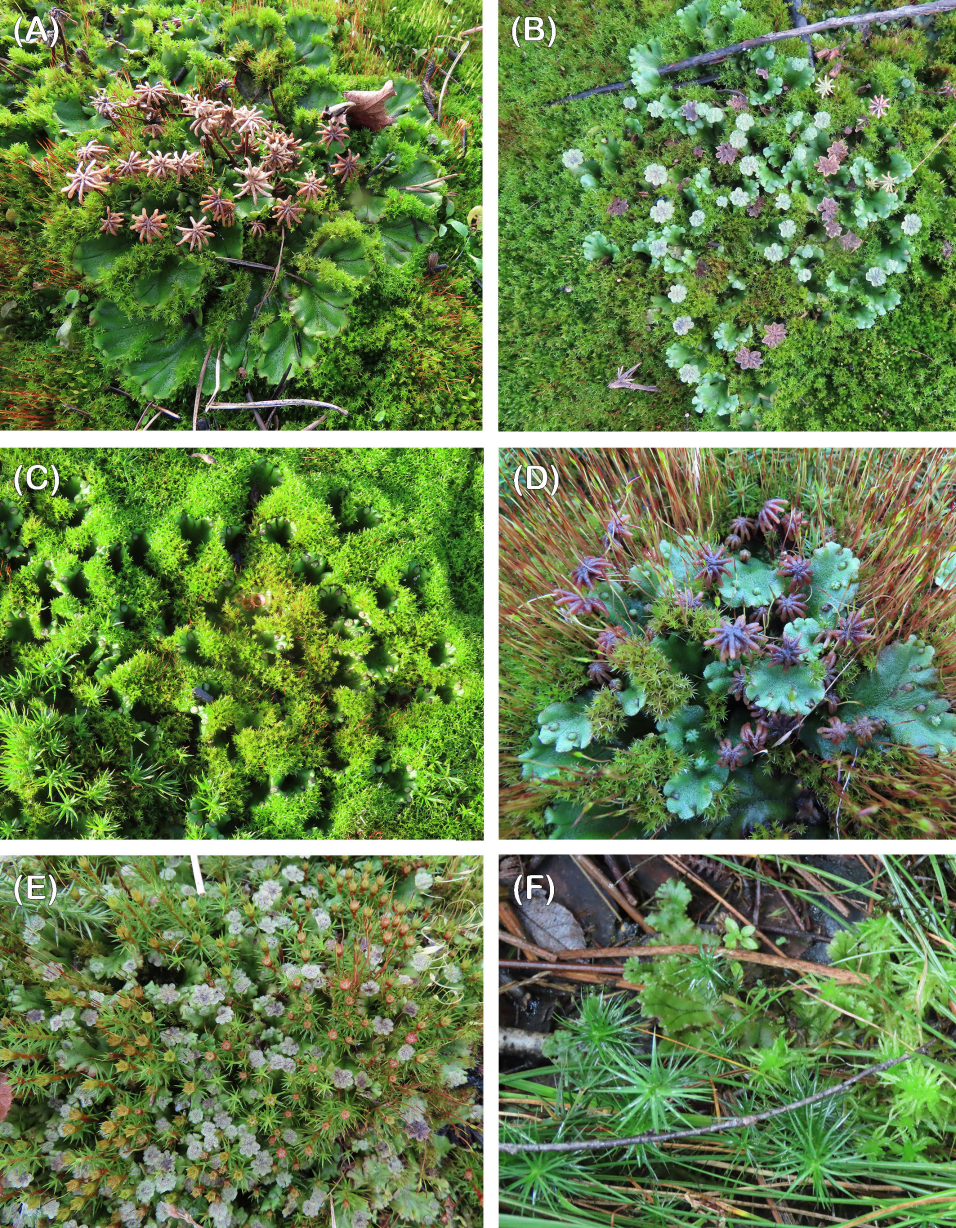
**(A)** Old females being overgrown by *Ceratodon.* Chobham, December 2021, **(B)** young and old males being overgrown by *Ceratodon.* Chobham, December 2021, **(C)** thalli almost completely overgrown by *Ceratodon.* Chobham, January 2022, **(D)** females with old and young archegoniophores surrounded by *Ceratodon* with spear stage sporophytes. Chobham, April 2022, **(E)** old and mature males being overgrown by male *Polytrichum juniperinum*, Chobham, April 2022, **(F)** vegetative *Marchantia* growing in a damp hollow with *Polytrichum commune* and *Sphagnum fimbriatum*. Chobham, September 2023.

The question of the ultimate disappearance of *Marchantia* from burnt site merits careful consideration. The reproductive biology of the liverwort followed much the same course after both the 2006 (Duckett and Pressel, 2009) and 2020 fires (this paper). After the 2006 Thursley fire it lasted until the winter of 2009; at both sites after the 2020 fires, it disappeared in the summer of 2022 following a severe drought. In general terms it is attributed to a fall in nutrient levels and competition at fire sites whereas persistence in urban sites is most likely due to additional nutrients from pollution, particularly NOx (Duckett and Pressel, 2016). Absence of mycorrhizal-like associations may also limit survival when nutrient levels fall (Rimington et al., 2020; Bowman et al., 2022); there is no interaction between *Marchantia* and the ericoid and *Cephaloziella* fungal endophytes, and *Cephaloziella* is long persistent after fires (Pressel et al., 2021; personal observations). It is also possible that shifts in soil bacterial and fungal communities (other than mycorrhizal) and/or in their interactions with *Marchantia*, may impact the establishment and ultimate demise of *Marchantia* post-fire. Recent research has highlighted the presence in *Marchantia polymorpha* subsp. *ruderalis* of several bacterial and fungal endophytes, with functions spanning the symbiotic spectrum, from beneficial to pathogenic (e.g. Alcaraz et al., 2018; Nelson et al., 2018; Poveda, 2024). These include bacterial genera capable of plant growth promotion, exudate degradation, nitrogen fixation, methylotrophs and disease-suppression (Alcaraz et al., 2018). *In vitro* experiments have demonstrated both beneficial and detrimental effects to *Marchantia* elicited by diverse fungal endophytes and that these effects could be shaped by nutrients available and the presence of other fungi (Nelson et al., 2018). As such, future research on the establishment and persistence of *Marchantia* post-fire should also consider this important aspect of its biology. *Marchantia* rapidly outcompetes *Funaria* with its short life cycle (**Figure 1B**) but may coexist for at least two years with *Ceratodon* (**Figures 6A-C**). Indeed, it can still reproduce sexually when overrun by this moss (**Figures 6B, D**). Ultimately *Polytrichum* spp. (**Figure 6E**) and *Campylopus introflexus* take over from these three species. These findings are one of the best examples to date of the detailed timing of a bryophyte succession.

Perhaps the most dramatic and surprising finding from our observations is the extinction of *Marchantia* caused by the severe drought in the summer of 2022 (Duckett et al., 2023). It has not reappeared at Thursley but recovered in a very damp site at Chobham associated with *Polytrichum commune* and *Sphagnum* spp. (**Figure 6F**). A dissection of the genes associated with desiccation tolerance across model bryophytes now beckons.

## Data availability statement

The original contributions presented in the study are included in the article/supplementary material. Further inquiries can be directed to the corresponding author.

## Author contributions

JD: Writing – original draft, Methodology, Investigation. SP: Writing – review & editing, Methodology, Investigation. JK: Writing – review & editing, Methodology, Investigation.
